# Selective adsorption of acidic gases from ternary mixture by acetate- and sulfonate-based ionic liquids at molecular level

**DOI:** 10.3906/kim-2103-34

**Published:** 2021-09-30

**Authors:** Sadiye VELİOĞLU

**Affiliations:** Gebze Technical University, Institute of Nanotechnology, Kocaeli, Turkey

**Keywords:** CO_2_ and H_2_S adsorption, ionic liquids, molecular dynamics simulations, adsorption mechanism

## Abstract

This paper attempts to elucidate the competitive adsorption mechanism of thin films of ionic liquids (ILs) on the surface of porous materials for acidic gases at a molecular level in order to design a proper material for the diminishment of gas emissions. Thin film 1-butyl-3-methylimidazalium ([BMIM]^+^) cation-based IL systems composed of four different anions such as [CH_3_CO_2_]^−^ and [CF_3_CO_2_]^−^ (acetate-based), and [CH_3_SO_3_]^−^ and [CF_3_SO_3_]^−^ (sulfonate-based) are created in contact with the gas phase containing ternary H_2_S/CO_2_/CH_4_:1/25/74 mixture. To define gas adsorption performance at gas–liquid interface and bulk liquid phase, classical molecular dynamics simulations are carried out. Adsorption of acidic gases is governed by the formation of an adsorbed gas layer on the surface of the ionic liquid based on thermodynamics aspects, and then the partial dissolution of gases in the bulk liquid phase is accompanied by the transport of gases. These behaviors are followed by several analysis methods in simulation approaches such as radial distribution function (RDF) of gases around specific atoms of ILs, lateral displacement of gas molecules, radial distance between gas and ILs, interaction energy between gases and ILs, and average number of hydrogen bonding between ions with and without adsorbed gases. Acetate-based ILs performed twice as good in CO_2_ adsorption capacity than sulfonate-based ILs. However, the one having –CF_3_ group in acetate-based ILs has short CO_2_ retention time and high CH_4_ adsorption capacity, diminishing the H_2_S+CO_2_/CH_4_ adsorption selectivity. High CO_2_ adsorption performance of acetate-based ILs is related to their strong anion–cation interaction and less hydrogen-bonding ability between cation tail and anion, which is the source for free space between anion and cation. Those with high adsorption capacities and long retention times are those that can ensure that CO_2_ molecules are coordinated in these free volumes between cation tails and anions. Therefore, here, the effect of different parameters on the CO_2_ and H_2_S adsorption over CH_4_ is revealed via atomistic design, and the importance of selection of suitable anions in IL for identifying potential nanocomposite adsorbent materials for acidic gas removal is highlighted.

## 1. Introduction

The global climate warming threatens ever-increasingly countries all around the world, which originates from the emission of greenhouse gases. Its consequences are started to damage our living environment and hinder economic development. Carbon dioxide (CO_2_) is the main responsible gas for the frightening state of affairs with its concentration in the atmosphere around 400 ppm [1]. Hydrogen sulfide (H_2_S) is the other acidic gas that threatens the environment and human health. Theemission of these acidic gases will be inevitable as long as we continue to use petroleum, coal, and natural gas as primaryglobal fuels. Additionally, natural sources such as volcanoes, natural gas wells, and sulfur springs, and industrial activitiesare the other sources for H_2_S. Therefore, CO_2_ and H_2_S storage and capture from fuels by chemical and physical absorptionand adsorption processes, or the combination of the features of absorbents and adsorbent processes are highly appreciateddue to their help in diminishing the acidic gas load in the atmosphere with low energy penalty [2]. Therefore, for theirmutual contributions, composite materials were produced by the incorporation of ionic liquid (IL) having permanentporosity into the porous solid adsorbent such as metal organic framework (MOF), covalent organic framework (COF), zeolite, etc. [3–4]. In this sense, superior acidic gas adsorption performance of ILs from biogas had led their widespreaduse in several separation processes by the impregnation in nanoporous solids, which is accepted as a promising strategy todesign new surface properties within porous materials for gas adsorption [5–8].

ILs composed of large asymmetric organic cations and smaller inorganic or organic anions are considered as green solvents due to their negligible vapor pressure and enhanced physical and chemical stability [9]. Their favorable solvation properties gained considerable attention in the processes of CO_2_ and H_2_S storage and capture. Since cation and anion of IL can be tailored, its physicochemical properties can be readily modified to optimize acidic gas absorption/adsorption performance. After the first discovery of CO_2_ adsorption capacity of imidazolium-based ILs by Blanchard et al. [10], they were started to be tested by several experimental and theoretical studies such as gravimetric microbalance [11], the synthetic (bubble point) method [12], and isochoric saturation [13] in experimental measurements as well as molecular dynamics (MD) [14], Monte Carlo (MC) [15], and quantum mechanical (QM) [16] simulations in theoretical computations. Since it was well-established that imidazolium-based ILs have high CO_2_ and H_2_S adsorption performances, their most suitable combinations with anions were intensely investigated. Within the hundreds of different anions, high acidic gas solubilities of acetate-based ILs [17–19] corresponding to their potential of formation of chemical bonds, and sulfonate-based ILs [20–22] at high pressures were revealed in the literature.

To further improve the separation properties of nanoporous materials, which are used as adsorbents, studies were concentrated on the impregnation of ILs into these porous materials. Mohamedali et al. reported an increase of 700 % for the [BMIM]^+^[Ac]^−^@ZIF-8 with 30 wt.% IL loading [5] and 200 % for [BMIM]^+^[Ac]^−^@HKUST-1 with 5 wt.% IL loading [6] in CO_2_ adsorption capacity compared to pristine MOFs at 303 K and 0.15 bar. Shahrom et al. [7] revealed that CO_2_ adsorption capacity of [DMEDAH]^+^[Ac]^−^@activated carbon (AC) enhanced from 0.08 mmol/g (pure AC) to 0.77, 0.87, and 0.36 mmol/g with 10, 20, and 30 wt.% IL loadings at 298 K and 1 bar. Similarly, Liu et al. [8] reported 80% improvement in CO_2_ adsorption capacities of polyetherimide (PEI)/SBA-15 composites with the impregnation of [EMIM]^+^[Ac]^−^ (from 1.18 to 2.20 mmol/g) at 303 K.

Considering high adsorption capacities of acetate- and sulfonate-based ILs for acidic gases, and their outstanding adsorption-based separation performances as impregnated into porous solids, the investigation of adsorption capacities of pristine acetate- and sulfonate-based ILs for the ternary mixture of CO_2_, H_2_S, and CH_4_ is emerged due to the determination of their actual performance. Since especially, the selective adsorption of H_2_S and CO_2_ from CH_4_ by ILs impregnated into different solid and polymeric materials is of great significance, it is required initially to understand the structure-property relationship of pristine ILs for acidic gas separation. Moreover, since there is a high demand on the identification of more realistic performance of adsorbents, we investigated H_2_S/CO_2_/CH_4_ ternary mixture separation by acetate- and sulfonate-based IL adsorbents. The most feasible and cost-effective way to carry out this analysis is to perform theoretical investigations using molecular dynamics simulations. Therefore, it is aimed in this study to scrutinize the adsorption mechanism of acetate- and sulfonate-based ILs for acidic gases in ternary mixture using molecular dynamics simulations. Four ILs consisting of same cation as 1-butyl-3-methylimidazalium [BMIM]^+^ and different anions such as alkylsulfonate [CH_3_SO_3_]^−^ (ASF), triflate [CF_3_SO_3_]^−^ (OTF), acetate [CH_3_CO_2_]^−^ (OAC), trifluoroacetate [CF_3_CO_2_]^−^ (TFA) are selected. The composition of ternary H_2_S/CO_2_/CH_4_:1/25/74 mixture was set following the acid gas content in raw natural gas streams, which vary according to the source, commonly in the range of 25–55 mol.% for CO_2_ and below 2 mol.% for H_2_S [23]. Since the highest CH_4_ concentration in the bulk phase will reduce the total adsorbed amount of CO_2_ and CH_4_ on any adsorbent material [24], the lowest content of CO_2_ (or highest content of CH_4_) is preferred in order to reveal the adsorption capacities of the selected ILs at the harsh conditions. It is worth to note that at high CO_2_ content or at high pressure, the adsorbed CO_2_ amount will increase.

## 2. Computational details

**Figure 1:**
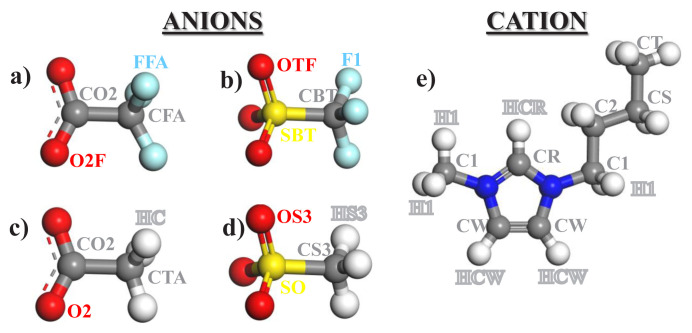
Molecular representation of anions such as (a) trifluoroacetate (TFA), (b) triflate (OTF), (c) Acetate (OAC), and (d) alkylsulfonate (ASF), and 1-butyl-3-methylimidazolium ([BMIM]^+^) cation with atomic site labels on each molecule. Note that hydrogen atoms having missing atomic sites in cation are labeled as HC.

Theoretical investigation is carried out using molecular dynamics simulations via Large-scale Atomic/Molecular Massively Parallel Simulator (LAMMPS) [25] open source software. Visual Molecular Dynamics (VMD) package [26] is used for the visualization of designed systems and post-analysis. For the ionic liquids (ILs) depicted in Figure 1, OPLS-AA [27] force field is used, but specific parameters developed by Lopes et al. [28–29] are adapted to encompass the entire homologous series of IL. A three-site rigid molecule with Lennard-Jones (LJ) 12-6 potential is used to model both CO_2_ [30] and H_2_S [31] and locations of partial point charges are set at the center of each side, as given in Table 1. Single-site spherical LJ 12-6 potential is used for CH_4_ [32] molecules. Long-range electrostatic interactions are treated with the PPPM Ewald method. Bond distances involving hydrogen atoms in IL are constrained using the SHAKE algorithm. The cut-off distance for non-bonded interactions is adjusted as 18 Å. The cross-term parameters for interaction between i and j atoms are calculated as ɛ_ij_=(ɛ_ii_×ɛ_jj_)^1/2^ and σ_ij_=(σ_ii_+σ_jj_)/2.

**Figure 2:**
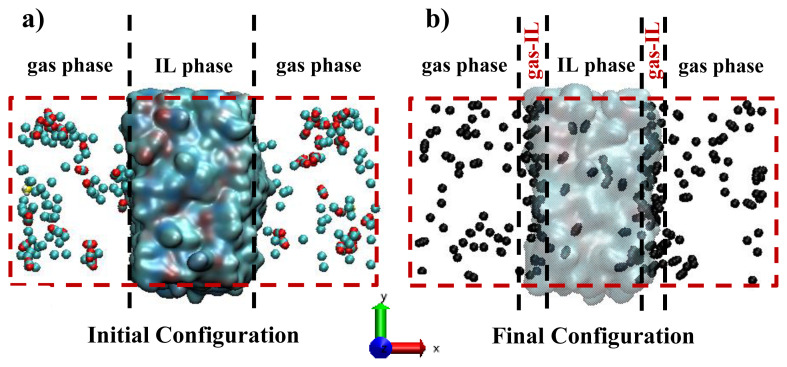
Representation of (a) initial and (b) final states of [BMIM]^+^[CH_3_CO_2_]^−^ system consisting of 500 ion pairs. Please note that randomly packed H_2_S, CO_2_, and CH_4_ molecules in (a) are represented with yellow, red and cyan colors, and all gas molecules in (b) are illustrated with black.

Figure 2 represents the initial state of one of the investigated simulation system containing IL film exposed to gas phase. As an example, the initial and final configurations of system of [BMIM]^+^[CH_3_CO_2_]^−^ are given in Figure 2 (a) and (b). Each system includes 500 ion pairs (namely, 500 anions and 500 cations) that constitute an IL layer of approximately 40 Å thicknesses along the x-axis. Initial configurations of systems including bulk IL layer and gas phase are randomly generated using the Packmol software [33]. Ion pairs consisting of IL are centered in the middle of unit-cell and surrounded with gas phases at both ends along the x-axis. The area of the IL surface contacting with the gas phase is about 64×64 Å^2^ (a side lengths of y- and x-axes), which is enough to observe a reliable statistic for the estimation of surface adsorption and interface region between IL and gas phases. Total thickness of gas phase along the x-axis is set to approximately 80 Å, including 2, 50, and 148 molecules of H_2_S, CO_2_, and CH_4_, respectively, which corresponds to the composition of the ternary H_2_S/CO_2_/CH_4_:1/25/74 mixture. Periodic boundary conditions are applied to each direction of simulation unit-cell.

First of all, initial configurations of ILs are allowed to relax with zero-temperature minimizations for 1 ns, followed by gradually increase to 500 K using NpT (iso-baric iso-thermal ensemble) runs for 5 ns, after which the temperature is gradually decreased to 298 within 2 ns. Subsequently, NpT run along 2 ns is performed at 298 K to equilibrate the system. Then, pre-equilibrated IL phase at 298 K is further used to generate a system such as that displayed in Figure 2 (a) by extending the length of unit-cell in x-direction and randomly distributing the gas molecules into this created vacuum phase to observe gas phase. Finally, in order to observe the equilibrated gas-IL interface, each system is relaxed using NVT (canonical ensemble) run for 5 ns at 298 K and 1 bar. For the sampling, further 15 ns NVT run is performed at the same conditions. For comparison, in each system, temperature is maintained at 298 K by the Nosé–Hoover thermostat with a coupling time constant of 100 fs, and pressure is maintained at 1 bar by the Nosé–Hoover barostat with a coupling time constant of 500 fs. The simulation time step is set to 1 fs. Figure 2 (b) displays the final state of well-equilibrated [BMIM]^+^[CH_3_CO_2_]^−^, which is observed after 5 ns of equilibration and 15 ns of sampling, revealing gas and IL phases and gas-IL interfaces, which is displayed with the two dashed lines.

Once the detailed atomistic systems are constructed, the conformational characteristics, namely, radial distribution function (RDF), interaction energy, and number of hydrogen-bonding (h-bonding) between each molecule types are estimated. The number of gas molecules within bulk IL layer and gas-IL interface, and distance between CO_2_ and specific groups on IL are calculated along the simulation time. Both density distribution and map of gases are provided to elucidate the adsorption mechanism of each IL for each gas. Finally, displacement of CO_2_ molecules adsorbed in the bulk of IL is provided by 3D maps.

**Figure 3:**
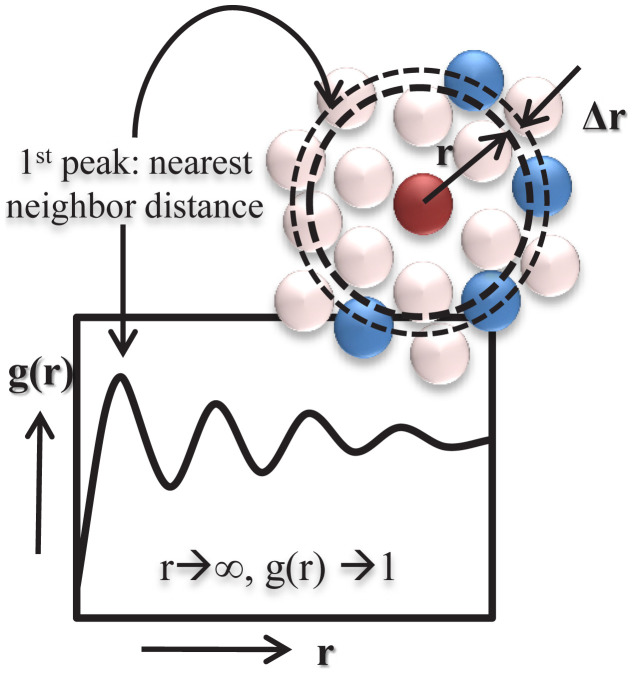
Schematic illustration of g(r) dependence on distance (r).

The RDF (g(r)) gives the probability of finding a given pair at defined distance compared with the average probability according to Equation 1.


(1) $$g_{ij}(r)=\frac{\Delta N_{ij}(r,r+\Delta r)}{4\pi r^{2}\Delta rN_{i}N_{j}}$$

where r is the distance between species i and j, ΔN_ij_(r, r+Δr) is the number of species j around i within a shell from r to r+Δr as given in Figure 3, N_i_ and N_j_ are the numbers of species i and j. According to the location and intensity of the peaks in the RDF, for instance, we can define the distribution of gas molecules around anion or cation of ILs.

H-bonding is defined based on the oxygen atoms in the anion of IL and carbon atoms in the cation of IL both in the presence and absence of gas molecules in the bulk IL phase. The applied criterion for h-bonding is that the distance between the donor and acceptor atoms is less than 3.2 Å and the angle of the C–H•••O bond is greater than 130°. Although it was well-demonstrated that the anion tends to orientate close to CR atom type (see Figure 1) in [BMIM]^+^ cation by creating h-bonding in the bulk IL phase [34], the numbers of h-bonding between anion and CW, C1, and CT atom types in the cation are also investigated.

The average interaction energy between defined pairs is extracted over simulation time to quantitatively investigate the interaction phenomena. Only inter-molecular (non-bonded) potential energy is involved in the interaction energy and averaged over time by 15 ns of the sampling for each IL system. The electrostatic interactions are modeled by the Coulombic potential using Equation 2, where the energy of the interaction is inversely proportional to the distance of separation r_ij_:


(2) $$E_{Coul.}=\frac{e^{2}}{4\pi {ɛ}_{0}}\sum_{i\neq j}\frac{q_{i}q_{j}}{r_{ij}}$$

The partial charges q_i_ and q_j_ are derived from quantum mechanics calculations, e is the charge of the electron, and ɛ_0_ is the dielectric permittivity of vacuum. Dispersive interactions are modeled with a 12–6 LJ potential with Equation 3:


(3) $$E_{LJ}=\sum_{i\neq j}{ɛ}_{ij}\left[{\left(\frac{\sigma_{ij}}{r_{ij}}\right)}^{12}-2{\left(\frac{\sigma_{ij}}{r_{ij}}\right)}^{6}\right]$$

ɛ_ij_ is the depth of the potential well, and σ_ij_ is the finite distance at which the inter-particle potential is zero, and they are empirical parameters derived from the fitting of the model to observed structural and physical property data.

Mass density profiles of each gas molecules, cation and anions, are computed for each chunk having 0.2 Å size (i.e. total-mass/volume) along the x-axis from the 15-ns sampling. Each system is split into uniform chunks perpendicular to the interface normal, and one-dimensional local density in each chunk along the x-axis of unit-cell is calculated in unit of molecule/Å^3^ as following:


(4) $$\rho (\text{x})=\frac{1}{{\text{A}}_{0}}\sum_{\text{i}=0}^{\text{N}}\delta (\text{x-}{\text{x}}_{\text{i}})$$

where ρ(x) is the global density profile, A_0_ is the nominal cross sectional area, N is the number of atoms, x_i_ is an atom coordinate along the axis perpendicular to the interface.

## 3. Results and discussion

### 3.1. Comparison of gas adsorption in each IL system

**Figure 4:**
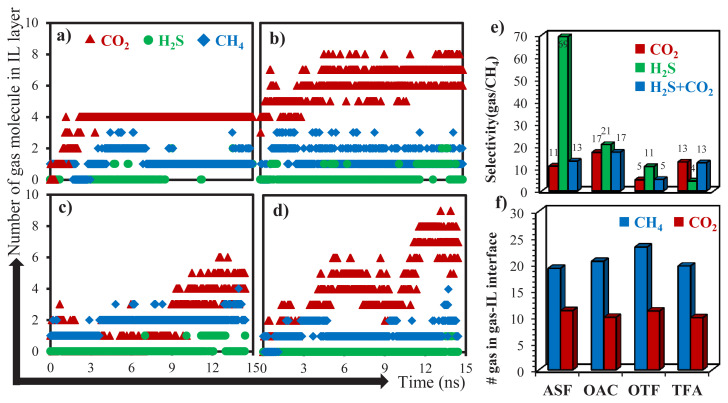
Adsorption performances of ILs for acidic gases from natural gas. Number of adsorbed gas molecules varied over 15-ns simulation time in the bulk phase of (a) ASF, (b) OAC, (c) OTF, and (d) TFA ionic liquids. (e) Adsorption selectivities of ILs for CO_2_/CH_4_, H_2_S/CH_4_, and H_2_S+CO_2_/CH_4_ mixtures, calculated using the average number of adsorbed gas molecules into the bulk phase of ILs within simulation time. (f) Average number of adsorbed CO_2_ and CH_4_ molecules on the gas-IL interfaces within simulation time by ILs.

To study the effect of pure ILs on acidic gas adsorption of bulk IL phase, number of adsorbed gas molecules varied within 15-ns simulation time is identified as given in Figures 4 (a-d).. Since high CO_2_ and H_2_S and less CH_4_ uptakes are desirable for the design of adsorbent material, initially the performance of ILs for CO_2_ and H_2_S are examined. Acetate-based ILs display higher CO_2_ adsorption capacity, almost double compared to the sulfonate-based ILs. However, when the adsorption rate of CO_2_ within 15-ns is considered, it can be seen that either acetate- or sulfonate-based ILs having –CH_3_ functional groups (ASF and OAC) reveal the frequent CO_2_ adsorption within 1–2 ns of their contact. But the ones having –CF_3_ functional groups display slow CO_2_ adsorption rate and large scattering in the number of adsorbed gas within the simulation time. On the other hand, it is not easy to provide similar conclusion when H_2_S adsorption of ILs is considered, where it follows the order of ASF > OAC ≈ OTF >> TFA. Since H_2_S adsorption of ASF is greater than the rest, it reveals highest H_2_S/CH_4_ adsorption selectivity. However, when CO_2_/CH_4_ selectivity of ASF is considered and the total acidic gas selectivity (H_2_S+CO_2_/CH_4_) is calculated, OAC takes the first place as shown in Figure 4 (e). Adsorption selectivity of OTF is the lowest due to having twice as much as CH_4_ adsorption compared to other ILs. Collectively, considering acidic gas separation performances, OAC is the best candidate within the investigated ILs with high adsorption capacity and rapid adsorption rate as well as adsorption selectivities of 17, 21, and 17 for CO_2_/CH_4_, H_2_S/CH_4_, and H_2_S+CO_2_/CH_4_, respectively. Finally, average numbers of adsorbed CO_2_ and CH_4_ molecules on both gas-IL interfaces (see Figure 2 (b)), whose thickness are varied between 6 and 8 Å, are given in Figure 4 (f). Although average number of CO_2_ located on the interface is almost same for the investigated ILs, there is a difference for those of CH_4_, even though not obvious. Highest CH_4_ adsorption of OTF in the bulk IL phase is evidently related to its high CH_4_ adsorption capacity on gas-IL interface. This result unfortunately hinders the adsorption selectivity of OTF and its potential use for acidic gas separation.

**Figure 5:**
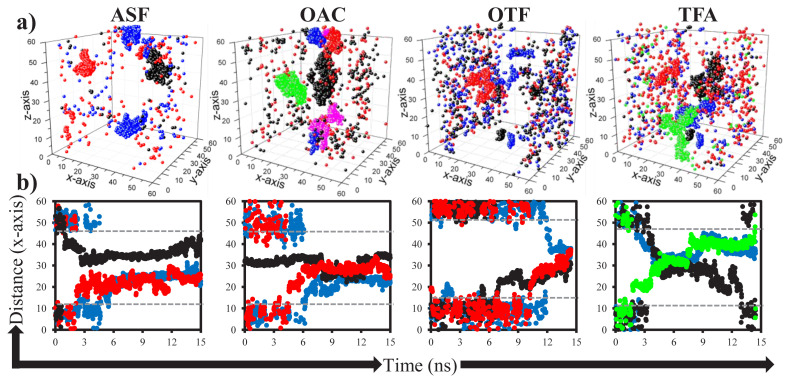
(a) Displacement of adsorbed CO_2_ molecules within the bulk IL phase along x-, y-, and z-axes. (b) Displacement of three adsorbed CO_2_ molecules, identified in (a) with the same color, along x-axis within 15-ns simulation time. Three, five, three, and four numbers of CO_2_ molecules are provided in (a) for ASF, OAC, OTF, and TFA, respectively. Grey dashed lines in (b) represents the approximate borders of bulk IL phase.

To reveal the mobility of adsorbed CO_2_ molecules into the IL phase, displacements of few CO_2_ molecules are illustrated by 3D maps in Figure 5 (a). The findings provided in Figures 4 (a-d) are also verified by Figure 5 (a) that adsorbed CO_2_ molecule on the surface of either ASF or OAC phases instantly prefers to move through the middle of bulk IL phase and keep trapped there. For OTF and TFA ILs, delay for the further move of CO_2_ through the IL bulk phase is yielded CO_2_ to hover over the gas-IL interface. After these adsorbed CO_2_ molecules diffused through the bulk IL phase, they revealed large displacement within the simulation time inside bulk OTF and TFA phases. However, the ones adsorbed by ASF and OAC ILs seem stuck in their location and display very little displacement. When the designed systems are considered (see Figure 2 (a)), IL phase is continuous in y- and z-axes, but finite in x-axis. Therefore, displacement of CO_2_ in x-axis from one interface to the other will provide information about the transport of adsorbed gases through IL phase. To reveal CO_2_ transport through IL phase, displacements of three CO_2_ molecules given in Figure 5 (a) along x-axis are provided in Figure 5 (b). The selected adsorbed three CO_2_ molecules are transported very rapidly through the middle of IL phase within approximately 3 ns for ASF and 6 ns for OAC, and stuck there until the end of simulation time (15 ns). This high adsorption and low transport rate (long retention time) of CO_2_ in OAC was also reported in the study of Aghaie et al. [35] where they compared [BMIM]^+^[CH_3_CO_2_]^−^ with [EMIM]^+^[BF_4_]^−^. For OTF, adsorption time of selected CO_2_ molecules is much longer than those of ASF and OAC. However, in addition to high adsorption capacity of TFA for CO_2_, CO_2_ transport rate in TFA phase is also greater than the rest of ILs. Two CO_2_ molecules which are adsorbed from one surface of TFA at around 3^rd^ ns released from the other surface of TFA at around 14^th^ ns. Additionally, CO_2_ molecules are not fixed in the same environment after the adsorbed by TFA, they jump from one region to the other, as illustrated by step-vise change in the displacement of x-axis in Figure 5 (b). Therefore, this shows that retention time of CO_2_ molecules is shorter in TFA, which is the other shortcoming of TFA in acidic gas separation in addition to its less H_2_S adsorption.

### 3.2. Structural differences in each IL system induced by CO_2_ adsorption

**Figure 6:**
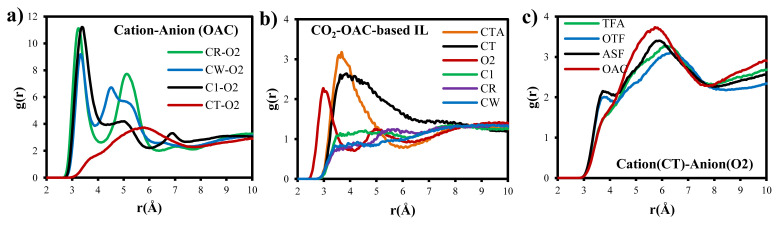
Radial density distribution functions between (a) specific atom sites in cation and oxygen in anion, (b) CO_2_ and specific atom sites in cation/anion, and (c) carbon atom of the tail of imidazolium cation and oxygen of the anion in each IL. Please note that (a) and (b) graphs are only provided for OAC IL due to the similarities in trend for other ILs.

In order to provide a mechanistic understanding of adsorption process induced by ILs, structural differences of IL systems observed after gas adsorption are compared. Orientation of oxygen atoms in anions with respect to the selected atoms in cation (see Figure 1 for atomic site labels) is investigated for each IL using RDF analysis as given in Figure 6 (a). Since the same trend in RDF of each IL is observed between anion and cation, only RDF of OAC is provided in Figure 6 (a). Oxygen atoms of anion dominantly prefer to locate around the carbon atoms in the polar head of cation, where this preference of several different anions to CR type carbon atom of imidazolium cation was supported in the literature [34, 36]. Klahn and Seduraman [37] proposed that CO_2_ adsorption led to reorientations of anions in the IL. However, similar with the data provided in this study, they also could not see any changes in RDFs of cation–anion pair when they compared RDFs observed before and after CO_2_ adsorption [37]. On the other hand, the most preferential adsorption sites of ion pairs for CO_2_ adsorption are displayed in Figure 6 (b) using RDF between CO_2_ and some specific atoms in ions. Again, since the high intensities in the RDFs of CO_2_–carbon atom in anion (e.g., CO_2_–CTA for OAC IL) and CO_2_–CT (carbon atom in the apolar tail of cation) are observed for all types of ILs, only RDF for OAC is provided. Figure 6 (b) evidently represents that CO_2_ molecules reveal high preference to the carbon atoms on tail of cation, since anions are loosely coordinated around them, as given in Figure 6 (a) with the peak having least intensity for cation (CT)–anion (O2) in OAC IL. Therefore, the RDFs of cation (CT)–anion (O2) in all IL systems observed after CO_2_ adsorption are compared in Figure 6 (c). The highest coordination of cation (CT) around anion (O2) is observed in OAC, and ASF follows it. This observation obviously explains the reason for their long retention time and low displacement of CO_2_ illustrated in Figure 5. Due to the comparably highly orientation of cation (CT) around anion (O2) in OAC and ASF, CO_2_ molecules adsorbed between them are not able to move freely and trapped in the free volume space existing between them, and, hence, cation–CO_2_–anion network dominates.

**Figure 7:**
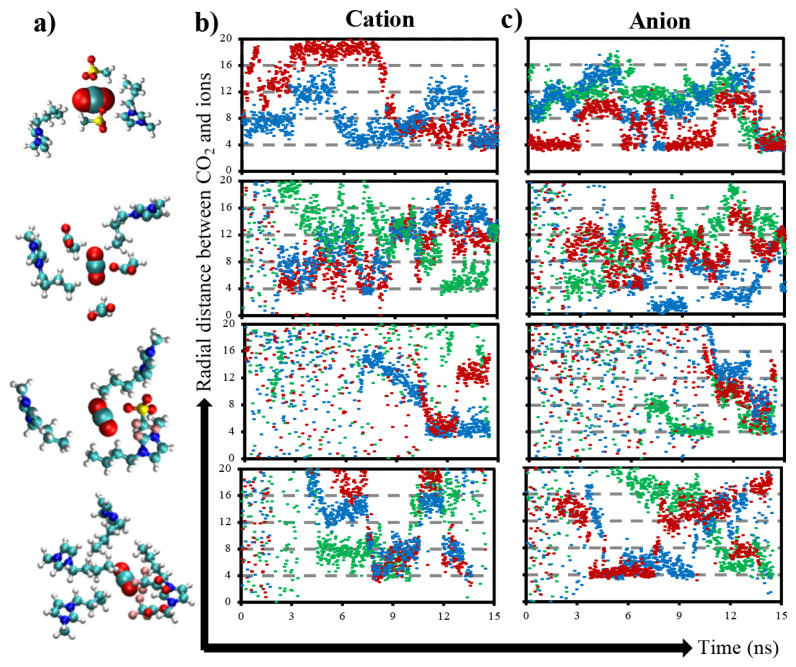
(a) Cation and anion orientations around one of the adsorbed CO_2_ molecule, where snapshots are taken from the last frame of 15-ns trajectory. Variation of radial distance between (b) randomly selected carbon atoms (CT) in the tail of cation and CO_2_ molecules, and (c) oxygen atoms of anion and CO_2_ molecules within 15-ns simulation time. Please note that from top to down, data belong to the ASF, OAC, OTF, and TFA, respectively.

The environment of adsorbed CO_2_ molecules as visualized in Figure 7 (a) is dominantly consisting of several cation tails and anions. Adsorbed CO_2_ molecules into ASF and OAC ILs are surrounded by almost same number of cations and anions during the simulation time. Only one frame at the end of simulation time is given in Figure 7 (a) which shows that adsorbed CO_2_ molecules into OTF and TFA ILs are surrounded by higher amount of cation tails than anions. This reveals the lack of network of cation–CO_2_–anion, which diminishes the trap of CO_2_ and, hence, enables easily transportation and desorption of CO_2_. On the other hand, Figures 7 (b-c) display the variation of radial distance between CO_2_ and the selected three cations and anions within simulation time, respectively. Accordingly, randomly selected adsorbed CO_2_ molecules into ASF and OAC do not change their environment within simulation time. When the distance between anion and CO_2_ (Figure 7 (c)) is compared with that of between tail of cation and CO_2_ (Figure 7 (b)), large variations exist due to the flexibility of anion and its small size. In the following section, this alteration of distance between CO_2_ and anion is supported by the interaction energy data.

**Figure 8:**
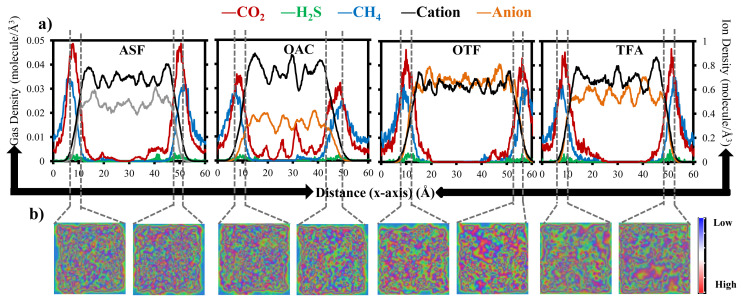
(a) Density profiles of CO_2_, H_2_S, CH_4_, anion, and cation along x-axis observed by averaging over 15-ns simulation time. Please note that dashed grey lines represent the borders of gas-IL interfaces. Please note that from left to right, data belong to the ASF, OAC, OTF, and TFA, respectively. (b) 2D density maps of ions on the surface of gas-IL interfaces, where blue and red regions represent the low and high ion densities, respectively.

In addition to the structure difference in the bulk IL phase, the difference in gas-IL interface is also important, which is the origin of adsorption process. Therefore, density distributions of each gas and ion along the x-axis of IL systems are given in Figure 8 (a). Density distributions of cations and anions within the bulk ASF and OTF IL phases are comparably smoother than those within OAC and TFA IL phases, which supports the homogeneous distribution of ions, lack of localization of anion-cation pairs and, hence, lack of empty space where CO_2_ can accommodate. However, although high adsorption capacity and large density variations of ions are observed for CO_2_ in OAC and TFA, CO_2_ density profile averaged from the ensemble of 15-ns simulation time displays that CO_2_ concentration within IL phase is only observed for OAC. Since retention time of CO_2_ within TFA IL phase is short, considerable density profile of CO_2_ could not be calculated. On the other hand, ion distribution maps on the lateral surface of gas-IL interface are provided in Figure 8 (b) in order to visualize the surface porosity and gain insight about its effect on gas adsorption. More uniform distributions of ions are available on the surface of ASF and OAC gas-IL interfaces. However, there are several indentations on both surfaces of OTF and TFA gas-IL interfaces, which hinder the anion–cation network and, hence, eliminate the fast CO_2_ adsorption. Probably, due to the fluorine groups, which hinder the rotation of anion, large cavities occurred on the surface of gas-IL interface of OTF and TFA systems. Collectively, coordination of anion around apolar tail of cation, anion–CO_2_–cation network, ratio of cation amount over anion surrounding CO_2_, and the surface porosity of gas-IL interface are the possible factors that are responsible for CO_2_ adsorption capacity of IL and retention rime within IL.

### 3.3. Interactions of ions with gases and within ion pairs

**Figure 9:**
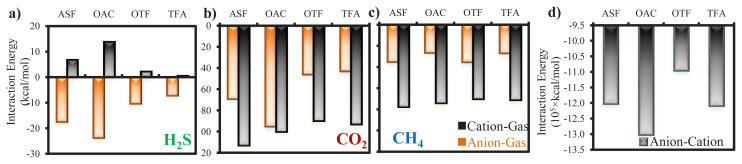
Average interaction energies over 15 ns between (a) anion–H_2_S, cation–H_2_S, (b) anion–CO_2_, cation–CO_2_, (c) anion–CH_4_, cation–CH_4_, and (d) anion–cation. Average interaction energies within gas–anion and gas–cation are represented in orange and black bars in (a – c).

On top of qualitatively visualizing the different affinities between gases and ILs in Figure 6 (b) and Figure 7, it is worthwhile to further quantify the interactions especially, since the potential interaction energies between each pairs may have different contributions to the adsorption of gases. Accordingly, the average interaction energies of H_2_S, CO_2_, and CH_4_ with cation and anion are quantified in Figures 9 (a-c), respectively. Cation molecule is the same in each IL and displays repulsive interaction energies with H_2_S regardless of anion type. When average numbers of adsorbed H_2_S molecules inside each IL bulk phase given in Figures 4 (a-d) are considered, highest and lowest values belong to ASF and TFA, respectively. Consistently, summation of average interaction energies of H_2_S–anion and H_2_S–cation pairs is greatest for ASF, which verifies its highest H_2_S adsorption capacity. Similarly, low H_2_S adsorption performance of TFA is related to low total interaction energy of H_2_S with both anion and cation (Figure 9 (a)). In the same manner, the highest CO_2_ adsorption performance of OAC can be explained with the highest total interaction energy existing with anion and cation, respectively. Additionally, although CO_2_ adsorption capacity of TFA, and average interaction energy existing between CO_2_ and cation for TFA are also close to those of OAC, CO_2_ molecules cannot be trapped within IL phase and desorbed from other surface of IL. This is evidently due to the low average interaction energy between CO_2_ and anion (Figure 9 (b)), proving that synergy within anion–CO_2_–cation is very important for CO_2_ adsorption process in ILs. On the other hand, similar adsorption capacities of ILs for CH_4_ are verified with the almost same average interaction energies of CH_4_ with both cation and anion, as given in Figure 9 (c). Finally, when average interaction energies between cation and anion for all ILs are considered in Figure 9 (d), CO_2_ adsorption performances of IL are compatible with them, where OAC and TFA reveal the greater values. Collectively, while cation–anion interaction is critical for adsorption of CO_2_, anion–CO_2_ interaction is the responsible one for the retention of CO_2_ within IL phase. Total gas–ion interactions are more dominant for H_2_S, which plays a role in the enhancement of H_2_S/CH_4_ selectivity for ILs, especially for ASF.

**Figure 10:**
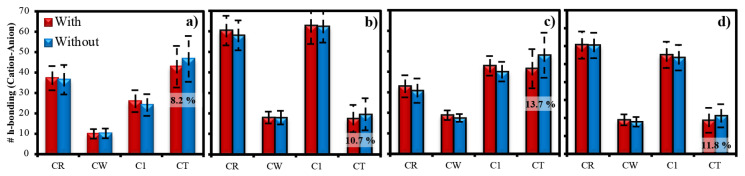
Average number of hydrogen bonding between specific groups (CR, CW, C1, CT) in cation and oxygen atoms in anion for (a) ASF, (b) OAC, (c) OTF, and (d) TFA ILs. Please see the investigated atomic sites in Figure 1, and note that error bars represent the standard deviation calculated in the last 5 ns of the production run when gases are present within IL (**with**), and in the final 2 ns of the production run when gases are absent within IL (**without**).

Figure 10 presents the number of h-bonding between cation and anion molecules averaged over last 5 ns for each system including adsorbed gases within IL phase, which supports the findings of RDF provided in Figure 6. To understand the contribution of h-bonding to the average total interaction energy between anion and cation, average number of h-bonding analyses are performed based on the criteria provided in the “Computational Details” section, for some of specific sites in cation (see Figure 1 for atomic site labels) with oxygen in anions in Figures 10 (a-d). Average number of h-bonding between CW and anion is the least in all ILs, providing the negligible effect of CW in anion–cation interaction. While the highest numbers of h-bonding for OAC and TFA exist within the pairs of CR–anion and C1–anion, those for ASF and OTF belong to the pair of CT–anion. It is represented in Figures 6 (c) and 7 that considerable amounts of CT are present around CO_2_ molecules for each IL systems. However, as seen in Figures 10 (b) and 10 (d), number of h-bonding between CT and anion in OAC and TFA, which are the systems having high CO_2_ adsorption capacities, is much lower than those of CR–anion and C1–anion. This reveals that since CT atoms are not capable of creating high amount of h-bonding with anions in OAC and TFA ILs, CO_2_ can easily find a free space around the tail of cation (CT) and adsorbed there. However, it is worth to mention that the main interaction between anion and cation, which is governed by CR and C1 atoms in the head group of cation, manages the network of cation–CO_2_–anion. Additionally, while there are similar standard deviations (error bars) in the number of h-bonding of CT in OAC and TFA compared to the other sites in cation, those are much larger in ASF and OTF. Instabilities in the number of h-bonding of CT with anion in ASF and OTF reveal their weakness, although their number of h-bonding is high compared to other identified sites. On the other hand, when average number of h-bonding between ions of ILs with and without gas molecules are compared in Figure 10, it is seen that only an increase in the h-bonding number is observed for the CT–anion pair, regardless of IL system, while those of other pairs in all ILs either slightly decrease, or they are almost similar. Since CO_2_ molecules prefer to locate between CT and anion, adsorption of CO_2_ leads to the orientation of anions towards CT atoms (tail of cation), and hence this enables the increase in the number of h-bonding due to the localization as shown in Figure 7 (a). Collectively, although CT sites do not have considerable effect on the strength of anion–cation network, they manage the CO_2_ adsorption by the help of h-bonding they created with anion.

## 4. Conclusion

The development of effective adsorbent material for acidic gas separation is of paramount importance to ensure low acidic gas emissions. Therefore, in this study, novel adsorbent materials as ionic liquids are investigated using molecular simulation approaches. It is aimed to provide a detailed knowledge of H_2_S, CO_2_, and CH_4_ adsorption capacities in gas-IL interface and bulk IL phase. Molecular dynamics simulations are used to reveal the adsorption mechanism of ASF, OAC, OTF, and TFA IL systems for acidic gases over 15 ns simulation time. Additionally, structural differences of each IL, their coordination around CO_2_, interaction between each pairs, and h-bonding between cation and anion are also identified for a detailed evaluation. OAC IL has the highest CO_2_ adsorption capacity inside bulk IL phase as well as highest CO_2_/CH_4_ adsorption selectivity compared to the rest of IL systems. However, greatest H_2_S/CH_4_ adsorption selectivity belongs to the ASF due to the highest H_2_S adsorption aroused from the strong interactions of H_2_S with anions and cations. Since there are uniform distributions of ions on gas-IL interface of ASF and OAC systems, fast CO_2_ adsorption is observed around 3 ns and fluctuate within the same value over the end of simulation time. On the other hand, although TFA reveals high CO_2_ adsorption capacity, it is not a prominent IL due to high CH_4_ and low H_2_S adsorption capacity led to the decrease in selectivities, and short retention time of CO_2_ inside IL bulk phase, which causes the adsorbed CO_2_ molecules to desorb on the other surface of IL phase. More importantly, it is revealed that the interaction energy of anion-cation governs the CO_2_ adsorption capacity by enabling anion-CO_2_-cation network. However, number of h-bonding between anion and cation should be less, so that the anion can be properly coordinated around the tail of cation to accommodate CO_2_ within the free space available between them. The attained information/data can assist to develop and design suitable IL-based adsorbents for acidic gas adsorption.

## Figures and Tables

**Table 1:** Potentials used for gas molecules in molecular simulations.

Gas molecule	Atom	ɛ (K)	σ (Å)	q(e)
CO_2_	C	27.0	2.80	0.70
	O	79.0	3.05	−0.35
H_2_S	H	3.9	0.98	0.124
	S	250	3.72	−0.248
CH_4_	single sphere	148.0	3.73	0.00
